# Unpacking cerumen impaction: a systematic review of clinical practice guidelines to support the development of the world health organization package of ear and hearing care interventions

**DOI:** 10.1186/s12875-026-03325-2

**Published:** 2026-04-28

**Authors:** Peter Kfoury, Keshav Shah, Lauren K. Dillard, Carolina Der, Pallavi Mishra, Rolvix H. Patterson, Mary Jue Xu

**Affiliations:** 1 Department of Otolaryngology - Head and Neck Surgery, University of Utah School of Medicine, 26 North Medical Dr., Rm 313, Salt Lake City, UT USA; 2 Global Otolaryngology-Head and Neck Surgery Initiative, Durham, NC USA; 3 Department of Otorhinolaryngology - Head and Neck Surgery, University of Pennsylvania, Philadelphia, PA USA; 4 Department of Otolaryngology - Head and Neck Surgery, Medical University of South Carolina, Charleston, SC USA; 5 Department of Noncommunicable Diseases, World Health Organization, Geneva, Switzerland; 6 Department of Head and Neck Surgery and Communication Sciences, Duke University School of Medicine, Durham, NC USA; 7 Hubert-Yeargan Center for Global Health, Duke University, Durham, NC USA; 8 Department of Otolaryngology-Head and Neck Surgery, University of California, San Francisco, CA USA

**Keywords:** Cerumen, Ear wax, Cerumen impaction, Systematic review, Practice guideline, World health organization

## Abstract

**Background:**

Cerumen impaction is a common, preventable cause of hearing loss that affects up to 30% of the global population. Despite its high burden, management practices vary widely, highlighting the need for comprehensive, evidence-based guidelines. This systematic review was conducted to inform the World Health Organization’s (WHO) Package for Ear and Hearing Care Interventions (PEHCI) by identifying and appraising high-quality clinical practice guidelines for cerumen impaction.

**Methods:**

A comprehensive search of bibliographic databases, guideline repositories, and organizational websites was conducted in August 2024. Guidelines published from 2014 onward were screened using predefined criteria. Eligible guidelines were assessed for quality using the AGREE II tool. Data were extracted using a standardized form based on the RIGHT checklist.

**Results:**

Out of 74 initially identified articles, two met all inclusion criteria and demonstrated sufficient quality: one developed by the American Academy of Otolaryngology – Head and Neck Surgery (AAO-HNS; total AGREE II score = 56.5) and the other by the UK’s National Institute for Health and Care Excellence (NICE; total AGREE II score = 62.5). Both guidelines emphasized patient education, symptom- and examination-based diagnosis, and use of cerumenolytics, irrigation, and manual removal for treatment. NICE discouraged manual syringes due to safety concerns, while AAO-HNS considered them a safe and acceptable treatment option when performed appropriately.

**Conclusion:**

This review highlights consistent, high-quality recommendations for cerumen impaction management and identifies areas of divergence relevant to global implementation. Findings will guide inclusion of cerumen impaction interventions in the WHO PEHCI and support the development of standardized, context-adaptable ear and hearing care services.

**Supplementary Information:**

The online version contains supplementary material available at 10.1186/s12875-026-03325-2.

## Background

Hearing loss affects 1.6 billion people worldwide, with projections estimating that 2.5 billion – one in four people - will experience some degree of hearing loss by 2050. The World Health Organization’s (WHO) 2021 World Report on Hearing highlighted the need to integrate ear and hearing care into universal health coverage to overcome disparities in access to and provision of hearing care services across all populations [1]. To support this aim, the WHO is developing an evidence-based package of ear and hearing care interventions (PEHCI) to help countries prioritize, budget, and incorporate hearing care into national health services, policies, and programs [2, 3]. This package will guide decision-making by identifying the most relevant ear and hearing care conditions and interventions necessary to address them across primary, secondary, and tertiary levels of care. Among these conditions, cerumen impaction has been identified as an underrecognized yet preventable and reversible cause of hearing loss [4, 5].

Cerumen impaction affects 5–30% of the global population and is a leading cause of primary care visits, particularly among individuals who are elderly, have disabilities, or have cognitive impairments [6–9]. Unaddressed cerumen can cause hearing loss, tinnitus, fullness, itching, otalgia, discharge, odor, and cough, while also preventing thorough assessment of the ear canal, tympanic membrane, and middle ear [6, 10]. Management approaches for this common condition vary considerably, both in clinical settings and through patient self-care. Epidemiologic surveys indicate that over 90% of individuals engage in ear cleaning practices for themselves or their children, such as inserting foreign objects (i.e., cotton-tipped swabs) [11–13]. These practices are often associated with increased risk of impaction, abrasions, perforations, and infections [11]. The high prevalence of unsafe ear-cleaning behaviors and their associated risks underscores the need for evidence-informed education and standardized clinical practices regarding safe management strategies.

While multiple resources for cerumen impaction interventions exist, they differ in scope, methodology, quality, and recommendations. Therefore, this systematic review assesses the quality of clinical practice guidelines (CPGs) and characterizes the current evidence on interventions for cerumen impaction to inform the development of the PEHCI.

## Methods

This systematic review was conducted in August 2024 in accordance with the Preferred Reporting Items for Systematic Reviews and Meta-Analyses (PRISMA) reporting guidelines and a predefined protocol (Appendix A1) [14]. CPGs were identified through a comprehensive search of bibliographic databases, guideline repositories, and professional organizations’ websites, along with a review of the reference lists of identified CPGs (Appendix A2).

Utilizing Covidence, a systematic two-stage screening process was implemented, consisting of title and abstract review followed by full-text assessment of potentially relevant guidelines. All articles were reviewed independently by two authors (KS, PK, RP, MX), with disagreements resolved by a third reviewer. Inclusion criteria required that CPGs were published between 2014 and 2024 and addressed the prevention, diagnosis, and/or management of cerumen impaction. The 2014 cutoff was selected to align with contemporary standards of guideline development and reporting. In addition to database searches, we conducted a targeted hand search for relevant guidelines published in Spanish, French, Chinese, and Russian using translated search terms. If non-English guidelines were identified, they were assessed for eligibility by speakers of those languages. Articles were excluded if they were commercially funded, had contributors with unmanaged conflicts of interest, or did not list affiliations. There were no restrictions on the age or location of patient populations.

Guidelines that passed full-text screening were then independently evaluated for quality by two authors (KS, PK) using the Appraisal of Guidelines for Research and Evaluation II (AGREE II) tool [15]. Based on criteria developed for the WHO Package of Eye Care Interventions and Package of Rehabilitation Interventions, CPGs were included if (1) the mean total score for nine selected items was at least 45 and (2) four key items each had a mean score of at least 3 (Appendix A3) [3, 16–19]. Finally, data were extracted from each included CPG using a standardized form adapted from the Reporting Items for practice Guidelines in HealThcare (RIGHT) checklist (Appendix A4).

### Role of the funding source

Funders of the study had no role in study design, data analysis, data interpretation, or writing of the report.

## Results

**Fig. 1:**
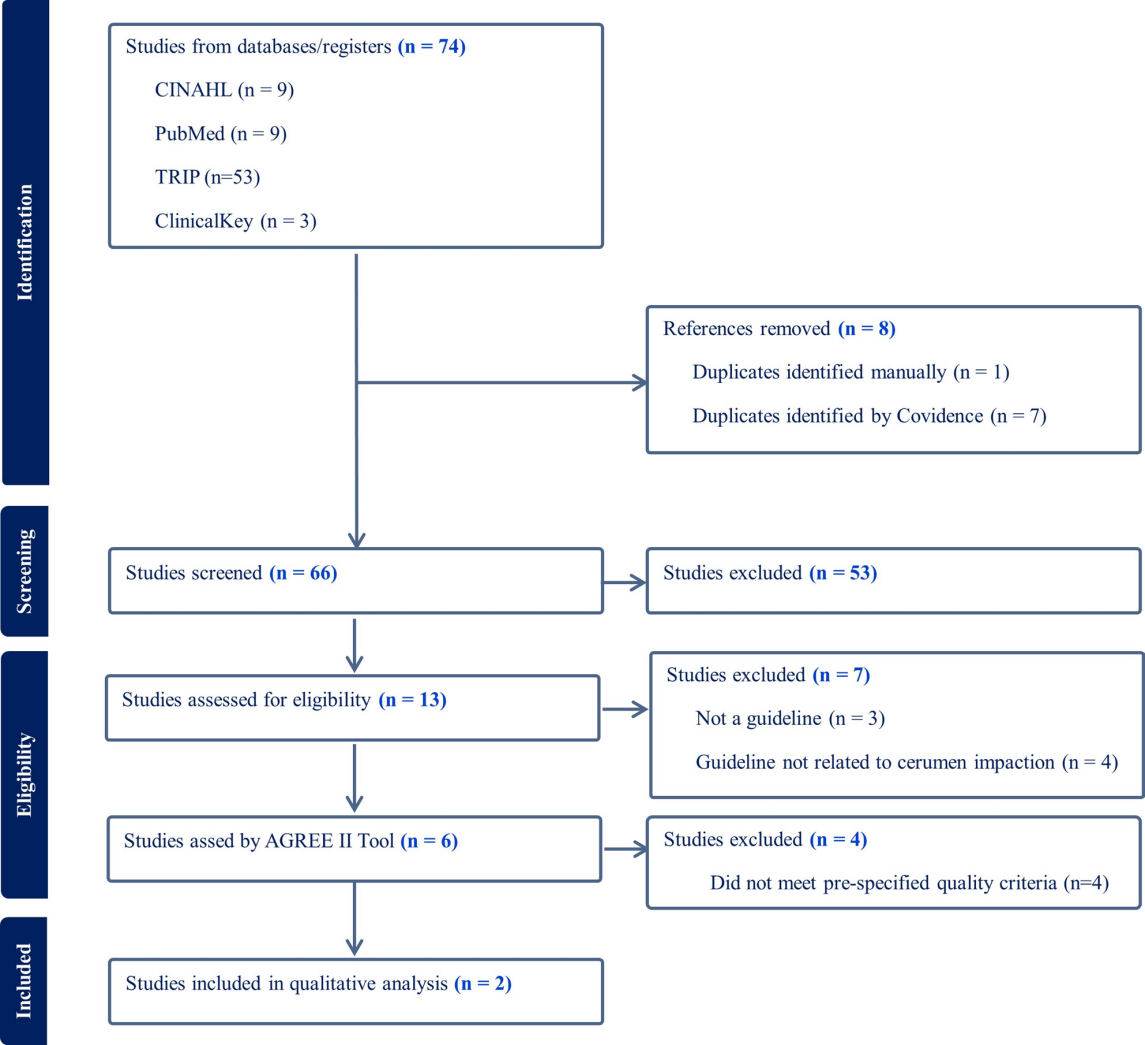
PRISMA flow chart depicting study selection process

The results of the comprehensive search are summarized in Fig. 1. The initial search yielded 74 articles. After removing duplicates and applying initial eligibility criteria, 66 titles/abstracts and 13 full-text articles were reviewed. After full-text review, six CPGs met the initial eligibility criteria and were assessed for quality using the AGREE II tool. Of these, four were excluded due to insufficient AGREE II scores (Table 1; Appendix A3). Two CPGs met al.l inclusion criteria and had the required mean score of *≥* 3 across the four key items and a mean total score of *≥* 45, as per the pre-defined threshold in the protocol for the development of the WHO PEHCI [3]. These CPGs were “Clinical Practice Guideline (Update): Earwax (Cerumen Impaction)” (2017), developed by the American Academy of Otolaryngology – Head and Neck Surgery (AAO-HNS) [10], and “Hearing loss in adults: assessment and management” (2018), developed by the National Institute for Health and Care Excellence (NICE) [20]. Subsequent recommendations and discussions are therefore restricted to these two guidelines.

**Table 1:** The AGREE II rating of guidelines after full-text screening

Guideline	Publication Year	AGREE II Ratings									
		Average of Key Items									Sum of Key Item Averages
		4	7	8	10	12	13	15	22	23	
AAO-HNS [10]	2017	7	6	7	5.5	7	4.5	7	7	7	58
NICE [20]	2018	7	7	5.5	7	7	6.5	7	7	7	61
Horton et al. [21]	2020	3	2.5	1	1.5	4.5	1	5.5	7	7	33
BSA [22]	2021	7	1	1	1.5	1	1.5	2.5	1	6.25	23
Oron et al. [23]	2015	3.5	1	1	1	1	1	1.5	1	1	12
Hauk et al. [24]	2017	1.5	1	1	1	1	1	1.5	1	1.5	10.5

After the full-text screening, the quality of the CPGs was evaluated using the Appraisal of Guidelines for Research and Evaluation (AGREE II) tool [15]. The nine key items (numbers 4, 7, 8, 10, 12, 13, 15, 22, and 23) from the AGREE II tool were used to evaluate the quality of the guidelines

*Abbreviations*: *AAO-HNS* American Academy of Otolaryngology – Head and Neck Surgery, *AGREE II* Appraisal of Guidelines for Research and Evaluation II, *BSA* British Society of Audiology, *NICE* National Institute for Health and Care Excellence

### Scope of CPGs

The CPG written by AAO-HNS describes the prevention and counseling, diagnosis, and management of cerumen impaction in patients over six months of age. This CPG does not apply to patients with dermatologic diseases of the ear canal, recurrent otitis externa, keratosis obturans, prior radiation therapy affecting the ear, exostoses or osteoma, neoplasms of the ear canal, previous tympanoplasty/myringoplasty, canal wall down mastoidectomy, or other surgery affecting the ear canal. The CPG by NICE describes the counseling and management of cerumen impaction in adults (> 18 years) with hearing loss, including those with pediatric-onset or congenital hearing loss. Both CPGs were aimed at patients and healthcare professionals at primary and secondary levels of care. Both CPGs used a multi-step process to assemble a multidisciplinary guideline committee that produced evidence-based recommendations that subsequently underwent external review by experts, public consultation, and peer review. Consensus recommendations are listed in Table 2. A full list of recommendations made by each CPG, separated by strength of evidence, is shown in Supplementary Table S1.

**Table 2:** Consensus recommendations

Prevention	Counselling	Clinicians should explain proper ear hygiene to prevent cerumen impaction when patients have an accumulation of cerumen.
		Advise adults not to remove earwax to clean their ears by inserting small objects, such as cotton buds, into the ear canal, as this could damage the eardrum and canal and push wax further down into the ear.
		Clinicians may educate/counsel patients with cerumen impaction or excessive cerumen regarding control measures, such as instilling prophylactic topical preparations, irrigating the ear canal, cleaning hearing aids, or routine cleaning of the ear canal by a clinician.
Diagnosis	Otoscopy	Clinicians should diagnose cerumen impaction when an accumulation of cerumen, as seen with otoscopy, is associated with symptoms and/or prevents needed ear assessment.
		Clinicians should identify patients with obstructing cerumen in the ear canal who may not be able to express symptoms (young children and cognitively impaired children and adults), and they should promptly evaluate the need for intervention.
		Clinicians should perform otoscopy to detect the presence of cerumen in patients with hearing aids during a health care encounter.
	Clinical History and Otoscopy	Clinicians should assess the patient with cerumen impaction by history and/or physical examination for factors that modify management, such as ≥ 1 of the following: anticoagulant therapy, diabetes mellitus, immunocompromised state, prior radiation therapy to the head and neck, ear canal stenosis, exostoses, or non-intact tympanic membrane.
Management	Ear wash, Instrumental Wax Extraction, Suction, and Cerumenolytic Agents	Clinicians should treat or refer to another clinician who can treat cerumen impaction when identified.
		Clinicians should treat, or refer to a clinician who can treat, the patient with cerumen impaction with an appropriate intervention, which may include ≥ 1 of the following: cerumenolytic agents, irrigation, or manual removal requiring instrumentation.
		Clinicians should not routinely treat cerumen in patients who are asymptomatic and whose ears can be adequately examined.
	Counselling	Clinicians should recommend against ear candling/coning.
	Cerumenolytic Agents	Clinicians may use cerumenolytic agents (including water or saline solution) in the management of cerumen impaction. No particular agent is superior to any other. Cerumenolytics should be avoided in patients with active infections of the ear canal.
	Ear Wash	Clinicians may use irrigation in the management of cerumen impaction, although it should be avoided in individuals who have a perforated TM or those who have had ear surgery.
	Instrumental Wax Extraction	Clinicians may use manual removal requiring instrumentation in the management of cerumen impaction.
Follow Up	Referral	If initial management is unsuccessful, clinicians should refer patients with persistent cerumen impaction to clinicians who have specialized equipment and training.
	Otoscopy, Otomicroscopy, and suction	Clinicians should assess patients at the conclusion of in-office treatment of cerumen impaction and document the resolution of impaction. If the impaction is not resolved, the clinician should use additional treatment. If full or partial symptoms persist despite resolution of impaction, the clinician should evaluate the patient for alternative diagnoses.

### Prevention and patient counseling

Both CPGs provided similar approaches to prevention and patient counseling. Patients should be advised against inserting objects into the ear canal for cerumen removal, as the ear is self-cleaning and because of the potential risks of increased infection, damage to the ear canal and/or tympanic membrane, and worsening cerumen impaction. Based on observational studies and risk-benefit analyses, clinicians were also advised to explain proper ear hygiene to prevent cerumen impaction.

The AAO-HNS CPG also provided clinicians the option to educate patients with cerumen impaction on control measures. Although this CPG acknowledges that empirical evidence was limited, it recommends several interventions to reduce cerumen impaction and prevent recurrence, including instilling prophylactic topical preparations, irrigating the ear canal, cleaning hearing aids, and routine clearing of the ear canal by clinicians. Physicians who choose to counsel patients accordingly should discuss the variety of topical preparations and devices for irrigation, acknowledge cost considerations, and allow for patient preference given the absence of clear evidence favoring one treatment approach over others.

### Diagnosis

The AAO-HNS CPG provided recommendations regarding the diagnosis of cerumen impaction, a topic not discussed by the NICE CPG. The AAO-HNS CPG supported otoscopy as the preferred diagnostic method to detect the presence of cerumen in patients. Cerumen impaction should be diagnosed when an accumulation of cerumen, as identified via otoscopy, is associated with symptoms (including otalgia, tinnitus, aural fullness, cough, and hearing loss) and/or prevents needed assessment of the ear (e.g., compromising auditory or vestibular testing, visualizing the tympanic membrane to assess the middle ear). Clinicians should also assess at-risk patients, including elderly adults with suspected dementia; developmentally delayed or nonverbal patients with behavioral changes; patients with hearing aids; and young children with fever, parental concern for cerumen impaction, or speech delay – even if the child does not explicitly identify or express symptoms. The approach to any patient with cerumen impaction should also include an assessment of potential complicating factors, including coagulopathies, diabetes mellitus, immunocompromise, history of radiation therapy to the head and neck, ear canal stenosis, exostoses, perforated tympanic membrane, and current otitis externa.

### Management

There were eight consensus recommendations from the CPGs regarding the management of cerumen impaction. Both the AAO-HNS and NICE CPGs strongly recommended that clinicians treat cerumen impaction when identified. The AAO-HNS CPG also recommended that patients should not be routinely treated if they have only partial cerumen occlusion of the external auditory canal, remain asymptomatic, and can undergo adequate otoscopic examination.

Both CPGs describe several methods to treat cerumen impaction. The AAO-HNS CPG recommends that cerumen impaction should be treated with cerumenolytic agents, irrigation, and/or manual removal using instrumentation. This CPG also provides considerations regarding age restrictions, length of treatment, precautions, benefits, risks and side effects, available resources, and provider experience as there are no comparative clinical trials addressing their relative efficacies. It further specifies that water is as effective as other cerumenolytic agents in treating cerumen impaction, noting that water should not be used for patients with otitis externa. The NICE CPG expands the array of management options, adding electronic irrigation, microsuction, and manual removal with probes and other instruments as appropriate approaches. It recommends these methods as long as the practitioner has appropriate training, the correct equipment is available, and there are no contraindications. Based on multiple studies demonstrating risk for serious injury and lack of efficacy, the AAO-HNS CPG recommends against ear candling for both the treatment and prevention of cerumen impaction.

The NICE CPG strongly recommends pretreatment cerumen softeners prior to ear irrigation in adults, either immediately before treatment or up to five days beforehand. The AAO-HNS CPG provides evidence regarding the utility of pretreatment, citing systematic reviews that suggest that administering cerumenolytics fifteen minutes before ear irrigation improves the efficacy of irrigation. However, it does not make any explicit recommendations regarding pretreatment. If irrigation is unsuccessful, the NICE CPG recommends that cerumen softeners are repeated or water is instilled in the ear canal for fifteen minutes prior to repeating ear irrigation. If this still does not work, NICE strongly recommends that patients are referred to specialist ear care services or an otolaryngologist for cerumen removal. In cases of persistent cerumen after unsuccessful initial management, the AAO-HNS CPG recommends referral to clinicians with specialized equipment and training.

There is one explicit contradiction between the AAO-HNS and NICE CPGs regarding the management of cerumen impaction. The AAO-HNS CPG recommends that aural irrigation can be performed either with a manual syringe or an electronic irrigator. The NICE CPG strongly recommends against the use of a manual syringe during aural irrigation due to variability in pressure applied to the external auditory canal and the risk of perforating the tympanic membrane. Although this CPG advises against manual syringing, it acknowledges that there is low-quality evidence comparing different treatment modalities and their associated risks. Furthermore, the NICE CPG concedes that the safety concerns pertain primarily to the use of large metallic syringes due to their capacity to apply high pressure. Therefore, this guideline implies that manual syringes could be used safely if adequate precautions are taken to avoid excessive pressure.

### Follow-up

Both CPGs recommend clinicians assess patients for the resolution of cerumen impaction at the conclusion of in-office treatment. The post-treatment otoscopic examination and symptom assessment should be documented in the medical record. If the cerumen impaction is not resolved, clinicians should provide additional treatment. This can include repeating the same treatment or trying an alternative method, such as manual removal if electronic irrigation was tried first or using cerumenolytics if they were not used initially. If symptoms persist despite resolution of cerumen impaction, clinicians should evaluate the patient for other possible underlying conditions.

## Discussion

This review systematically identified and appraised existing CPGs for the prevention, diagnosis, and management of cerumen impaction. Although several guidelines were identified through the comprehensive search, only two satisfied the inclusion criteria and demonstrated sufficient methodological quality based on the AGREE II instrument [3, 15]. These high-quality guidelines – authored by AAO-HNS and NICE – showed strong consensus on key aspects of the management for cerumen impaction for individuals aged 6 months and older. Both CPGs emphasize the importance of removing cerumen when it causes symptoms or prevents necessary examination, advise against inserting objects into the ear canal for cleaning, acknowledge multiple methods for cerumen removal (including cerumenolytics, irrigation, and manual removal), and recommend reassessment after treatment. Their broad applicability across age groups and endorsement of diverse treatment approaches provide flexibility for global implementation, allowing for adaptation based on available resources, provider training, and patient preferences. This systematic review will inform cerumen impaction interventions and necessary resources included in the WHO PEHCI, supporting countries and other stakeholders with the selection of priority EHC interventions to incorporate into national health services packages and policies.

Many of the recommendations within the included CPGs are derived from expert consensus rather than high-quality evidence, reflecting the limited availability of randomized or comparative studies on cerumen removal methods. Indeed, the discrepancy between CPG recommendations regarding the use of manual irrigation deserves particular attention in the global context. While the NICE CPG strongly recommends against using manual syringes due to concern about non-standardized pressure, this recommendation emerged from lower-quality evidence. Importantly, the guideline acknowledges that safety concerns derive from historical complications associated with outdated metal syringes. The NICE guideline explicitly states that “ear syringing with a large metal syringe or similar obsolete equipment is potentially harmful” and that “irrigation using an electronic ear irrigation machine which pumps water into the ear at a controlled pressure is safer” [20]. Our interpretation of this statement is that the NICE recommendation was based on concerns related to outdated metal syringes with uncontrolled pressure – that could potentially cause a tympanic membrane perforation – rather than on direct evidence against the safe use of modern syringes when performed with appropriate precautions. This distinction is especially relevant for low- and middle-income countries, where electronic irrigators may not be accessible or affordable, making plastic syringes the most practical option. The substantial cost difference between manual syringes (approximately $1–5 USD) and electronic irrigators ($40–100 USD) cannot be ignored when making resource-conscious decisions [25]. With appropriate risk mitigation strategies – including proper training on using a safe amount of pressure, using body-temperature water, and ensuring clear visualization throughout the procedure – manual syringes may offer a viable option in resource-constrained settings. However, further research is needed to optimize their safety and effectiveness in these settings.

Implementation of these guidelines may face challenges in resource-constrained settings, where the burden of preventable hearing loss is highest and significant barriers – such as limited availability of providers, transportation to facilities with hearing care, and knowledge about hearing care – are common [26]. Implementation science offers valuable frameworks to adapt and scale evidence-based interventions in such contexts, focusing on pragmatic outcomes such as feasibility, acceptability, fidelity, and sustainability [27, 28]. These approaches should be prioritized in future work (e.g., guideline development and implementation, adaptation to other settings) to address challenges, including limited access to training (including simulation strategies) and essential equipment (i.e., otoscopes), which can prevent even basic cerumen assessment and management [29]. Potential solutions include task-sharing strategies, where trained non-specialist health workers perform appropriate management of cerumen impaction, and approaches that reduce reliance on specialized equipment, such as cerumen softening with water or readily available cerumenolytics [30]. Research is also needed to develop and evaluate service delivery models that incorporate cerumen management in primary care, delineate cerumen management competencies across health worker cadres, and create standardized quality assurance mechanisms. In recent years, low-cost smartphone otoscope attachments and speculum adapters have become increasingly accessible and may represent a pragmatic solution in low-resource environments where conventional otoscopes are unavailable. These devices – when paired with smartphones – could facilitate basic ear visualization, safer cerumen management, and image capture for training and/or remote supervision [31].

This systematic review has several limitations. Although our search identified six CPGs on cerumen impaction, only two met the methodological standards for inclusion in this review. This could limit the scope of the analysis, but it ensures that the resulting recommendations are of high quality. Both guidelines were developed in high-income settings (US and UK) and did not address implementation considerations for resource-constrained settings, potentially limiting their generalizability. To address this limitation, the development of the PEHCI will engage a diverse group of experts and stakeholders from all six WHO regions and from countries across all income levels, helping to ensure that the selected interventions are globally relevant, feasible, and adaptable to a range of healthcare systems. Additionally, the stringent quality requirements imposed by the AGREE II tool may have excluded guidelines that, while methodologically imperfect, contain valid and useful recommendations based on clinical expertise. The lack of randomized trials comparing removal methods, acknowledged by both guidelines, also limits evidence-based conclusions on treatment superiority for cerumen impaction and highlights the need for further research. Finally, both guidelines provided limited guidance on the applicability of recommendations to infants under six months of age, individuals with significant comorbidities, or those with a history of ear surgery.

Looking forward, research should address several gaps identified in this review. High-quality comparative effectiveness studies of cerumen impaction management approaches are needed. In particular, outcomes important to patients should be evaluated, such as comfort, preference, and resolution of symptoms. Cost-effectiveness analyses comparing different approaches would allow health systems to make evidence-based resource allocation decisions. Lastly, studies in special populations, particularly children and those with cognitive impairment, would address essential gaps in the current evidence base.

## Conclusion

Cerumen impaction is a prevalent, preventable, and treatable condition that contributes to reversible hearing loss, aural fullness, discomfort, tinnitus, cough, and interference with diagnostic assessment of the ear. This review identified and evaluated two high-quality CPGs that emphasize the need for accurate diagnosis, symptom-driven management, and patient education on safe aural hygiene. These findings provide a foundation for integrating cerumen management into the WHO PEHCI. Cerumen impaction represents “low-hanging fruit” in addressing preventable hearing loss globally; with modest resources and quality training, its burden can be substantially reduced across diverse healthcare settings. Looking forward, future guideline development and research efforts should prioritize a comparative evaluation of the effectiveness of management tools available for cerumen impaction removal.

## Supplementary Information

Supplementary Material 1. Complete list of official recommendations published by qualifying CPGs, delineated by strength of recommendation and author.

Supplementary Material 2. Appendix A1-A4.

## Data Availability

The datasets generated or analyzed during the current study are available from the corresponding author on reasonable request.

## Abbreviations

AAO-HNS: American Academy of Otolaryngology – Head and Neck Surgery
AGREE II: Appraisal of Guidelines for Research and Evaluation II
CPG: Clinical Practice Guideline
NICE: National Institute for Health and Care Excellence
PEHCI: Package for Ear and Hearing Care Interventions
PRISMA: Preferred Reporting Items for Systematic Reviews and Meta-Analyses
RIGHT: Reporting Items for Practice Guidelines in HealThcare
USD: United States Dollar
WHO: World Health Organization
