# Supplementary material for: Unpacking cerumen impaction: a systematic review of clinical practice guidelines to support the development of the world health organization package of ear and hearing care interventions
Source: BMC Prim Care. 2026 Apr 28;27:170. doi: 10.1186/s12875-026-03325-2 (PMC13130458; doi:10.1186/s12875-026-03325-2)
Supplement: Supplementary file 1 — Supplementary Material 1. Complete list of official recommendations published by qualifying CPGs, delineated by strength of recommendation and author. [file 12875_2026_3325_MOESM1_ESM.docx]

*Supplementary Table 1: Complete list of official recommendations published by qualifying CPGs, delineated by strength of recommendation and author.*

|  | **AAO-HNS 2017** | **NICE 2018** |
| --- | --- | --- |
| **Strong Recommendations** | - Clinicians should treat, or refer to another clinician who can treat, cerumen impaction when identified. | - When carrying out ear irrigation in adults, use pretreatment wax softeners, either immediately before ear irrigation or for up to 5 days beforehand. If irrigation is unsuccessful, repeat use of wax softeners or instill water into the ear canal 15 mins before repeating ear irrigation. - If ear irrigation is unsuccessful after second attempt, rear to specialist ear care service or ENT for earwax removal. - Do not offer adults manual ear syringing to remove earwax. |
| **Recommendations** | - Clinicians should explain proper ear hygiene to prevent cerumen impaction when patients have an accumulation of cerumen. - Clinicians should not routinely treat cerumen in patients who are asymptomatic and whose ears can be adequately examined. - Clinicians should identify patients with obstructing cerumen in the ear canal who may not be able to express symptoms (young children and cognitively impaired children and adults), and they should promptly evaluate the need for intervention. - Clinicians should perform otoscopy to detect the presence of cerumen in patients with hearing aids during a health care encounter. - Clinicians should treat, or refer to a clinician who can treat, the patient with cerumen impaction with an appropriate intervention, which may include ≥1 of the following: cerumenolytic agents, irrigation, or manual removal requiring instrumentation. - Clinicians should recommend against ear candling/coning for treating or preventing cerumen impaction. - Clinicians should assess patients at the conclusion of in-office treatment of cerumen impaction and document the resolution of impaction. If the impaction is not resolved, the clinician should use additional treatment. If full or partial symptoms persist despite resolution of impaction, the clinician should evaluate the patient for alternative diagnoses. - If initial management is unsuccessful, clinicians should refer patients with persistent cerumen impaction to clinicians who have specialized equipment and training to clean and evaluate ear canals and tympanic membranes. - Clinicians should diagnose cerumen impaction when an accumulation of cerumen, as seen with otoscopy is associated with symptoms and/or prevents needed assessment of the ear. - Clinicians should assess the patient with cerumen impaction by history and/or physical examination for factors that modify management, such as ≥1 of the following: anticoagulant therapy, diabetes mellitus, immunocompromised state, prior radiation therapy to the head and neck, ear canal stenosis, exostoses, nonintact tympanic membrane. | - Consider ear irrigation using an electronic irrigator, microsuction, or another method of earwax removal (ex. manual removal with a probe) for adults in primary or community ear care services if the practitioner has training/expertise in the method and is aware of contraindications to the method, and if the correct equipment is available. - Advise adults not to remove earwax to clean their ears by inserting small objects, such as cotton buds, into the ear canal, as this could damage the eardrum and canal and push wax further down into the ear. |
| **Weak Recommendations** | - Clinicians may use cerumenolytic agents (including water or saline solution) in the management of cerumen impaction. No particular agent is superior to any other. Cerumenolytics should be avoided in patients with active infections of the ear canal. - Clinicians may use irrigation in the management of cerumen impaction, although it should be avoided in individuals who have a perforated TM or those who have had ear surgery. - Clinicians may use manual removal requiring instrumentation in the management of cerumen impaction. - Clinicians may educate/counsel patients with cerumen impaction or excessive cerumen regarding control measures, such as instilling prophylactic topical preparations, irrigating the ear canal, cleaning hearing aids, or routine cleaning of the ear canal by a clinician. | - None |
