# Supplementary material for: Unpacking cerumen impaction: a systematic review of clinical practice guidelines to support the development of the world health organization package of ear and hearing care interventions
Source: BMC Prim Care. 2026 Apr 28;27:170. doi: 10.1186/s12875-026-03325-2 (PMC13130458; doi:10.1186/s12875-026-03325-2)
Supplement: Supplementary file 2 — Supplementary Material 2. Appendix A1-A4. [file 12875_2026_3325_MOESM2_ESM.docx]

**APPENDIX A1**

*Adapted from the WHO’s “Protocol for the development of the WHO Package of Ear and Hearing Care Interventions” [1].*

Overview

Clinical practice guidelines (CPGs) will be the main source of evidence used to identify evidence-based ear and hearing care (EHC) interventions. CPGs are highly valuable because they (1) cover the continuum of clinical care for a given ear or hearing condition, (2) combine scientific evidence with clinical expertise, and (3) cover evidence gaps of published literature through expert consensus procedures.

Identification of CPGs

A systematic literature search of guideline databases and selected academic databases will be conducted. Websites for professional otolaryngology and audiology associations will also be searched for relevant guidelines. As necessary, search terms and filters will be adapted to effectively conduct the search across different databases and websites. Searches will identify CPGs published in the last 10 years (from 2014 onwards) and in the English language. In addition to database searches, we will conduct a targeted hand search for relevant guidelines published in Spanish, French, Chinese, and Russian using translated search terms.

Two authors will serve as reviewers and will independently screen the titles and abstracts of the CPGs. The following exclusion criteria will be applied: (1) the identified literature is not a guideline, (2) the guideline is not related to the selected EHC condition, and (3) the guideline was published prior to 2014. Full-text screening of the remaining CPGs will also be conducted independently by two reviewers. Guidelines will be excluded if there is a (1) presence of commercial funding and/or unmanaged conflicts of interest of contributors; and/or (2) absence of affiliations of all contributors. Any disagreements related to inclusion/exclusion of guidelines will be resolved by a discussion between the two reviewers and will involve a third reviewer as needed.

After the full-text screening, the same reviewers will independently evaluate the quality of the CPGs using the Appraisal of Guidelines for Research and Evaluation (AGREE II) tool [2]. Nine items (numbers 4, 7, 8, 10, 12, 13, 15, 22 and 23) from the AGREE II tool will be used to evaluate the quality of the guidelines [1,3,4]. For a given guideline, if the rating of an item differs by more than two points between the two members/experts, they will discuss results and involve a third reviewer, as needed, to reach consensus. Guidelines will be excluded if the members/experts rate the average score for items 4, 7, 8, 12, or 22 below 3 points, and if the sum of the average score for all nine items is less than 45 points [3].

A maximum of five CPGs will be selected for each EHC condition. If there are guidelines for specific age groups for a given condition (e.g., children, youth, adults), up to 5 CPGs will be selected for each age group. If more than 5 guidelines are identified for a given EHC condition, the selection of CPGs will be determined via consensus procedures among members of the WHO EHC Programme (with or without input from technical working group members). A consensus will be achieved by considering relevant information, including AGREE II scores, date of publication, and representativeness of the CPG, in terms of country, regional, or international scope of the guidelines.

Data Extraction and Preparation

One reviewer (KS or PK) will complete data extraction, which will be checked by a second reviewer (MX or RP). For CPGs, data extraction will include the following: (1) information on the guideline (i.e., title, authors, year of publication); (2) reference to the recommendations, interventions, and related outcomes; (3) content and strength of the recommendations; and (4) quality of evidence related to the recommendations.

**APPENDIX A2**

**Bibliographic Databases**

- PubMed: 9 results
  - Search string: ("guideline*"[Title/Abstract] OR "clinical protocol*"[Title/Abstract] OR "standard of care"[Title/Abstract]) AND ("cerumen impaction"[Title/Abstract] OR "impacted earwax"[Title/Abstract])
  - Filters: 2014 - current
- CINAHL: 9 results
  - Search string: (AB guideline* OR AB “clinical protocol*” OR AB “standard of care”) AND (AB cerumen impaction OR AB impacted cerumen)
  - Filters: 2014 - current
- Clinical Key: 3 results
  - Search string: “cerumen impaction”
  - Filters: Guidelines
- TRIP: 53 results
  - Search string: (cerumen impaction)
  - Filters: Guidelines, 2014 - 2024
- Global Index Medicus: 0 results
  - (cerumen impaction)
  - Filters: Practice guidelines, 2014-2024

**Guideline Repositories**

- [ECRI Guidelines Trust](https://guidelines.ecri.org/): 0 guideline
- [Guideline Central](https://www.guidelinecentral.com/guidelines/): 19 guidelines (2 duplicates and the rest excluded based on title)
- [Guidelines International Network](https://guidelines.ebmportal.com/): 1 guideline (duplicate)

**Professional Organizations’ Websites**

- [American Academy of Audiology](https://www.audiology.org/): 0 guidelines
- [American Academy of Family Physicians](https://www.aafp.org/family-physician/patient-care/clinical-recommendations.html): 0 guidelines
- [American Academy of Otolaryngology - Head and Neck Surgery](https://www.entnet.org/): 1 published guideline
- [American Speech-Language Hearing Association](https://www.asha.org/): 0 guidelines
- [Audiology Australia](https://audiology.asn.au/standards-guidelines/): 1 unpublished guideline
- [British Academy of Audiology](https://www.baaudiology.org/professional-information/nhs-and-healthcare-publications/#block3): 0 guidelines
- [British Society of Audiology](https://www.thebsa.org.uk/guidance-and-resources/current-guidance/): 1 published guideline
- [European Federation of Audiology Societies](https://efas.ws/): 0 guidelines
- [Indian Council of Medical Research](https://main.icmr.nic.in/): 0 guidelines
- [National Institute for Health and Care Excellence](https://www.nice.org.uk/guidance/conditions-and-diseases): 0 guidelines
- [South African Speech Language Hearing Association](https://saslha.co.za/): 0 guidelines
- [World Health Organization](https://www.who.int/publications/i?publishingoffices=c09761c0-ab8e-4cfa-9744-99509c4d306b): 0 guidelines

**APPENDIX A3**

Quality assessment of CPGs using the AGREE II tool. Items with two asterisks (**) were considered key items. Only two CPGs (NICE 2018, Schwartz 2017) had the required mean score of >3 in the four key items and mean total score of ≥45.

| **Author, year** | **4** | **7**** | **8**** | **10** | **12**** | **13** | **15** | **22**** | **23** | **Sum** |
| --- | --- | --- | --- | --- | --- | --- | --- | --- | --- | --- |
| **NICE, 2018 [5]** | 7 | 7 | 7 | 7 | 7 | 6.5 | 7 | 7 | 7 | 62.5 |
| **Schwartz, 2017 [6]** | 7 | 6 | 5.5 | 5.5 | 7 | 4.5 | 7 | 7 | 7 | 56.5 |
| **Horton, 2020 [7]** | 3 | 2.5 | 1 | 1.5 | 4.5 | 1 | 5.5 | 7 | 7 | 33 |
| **BSA, 2021 [8]** | 7 | 1 | 1 | 1.5 | 1 | 1.5 | 2.5 | 1 | 6.5 | 23 |
| **Oron, 2015 [9]** | 3.5 | 1 | 1 | 1 | 1 | 1 | 1.5 | 1 | 1 | 12 |
| **Hauk, 2017 [10]** | 1.5 | 1 | 1 | 1 | 1 | 1 | 1.5 | 1 | 1.5 | 10.5 |
| Abbreviations: NICE, National Institute for Health and Care Excellence; BSA, British Society of Audiology | | | | | | | | | | |

Excerpts from the AGREE II Tool’s User’s Manual are reproduced below to summarize the criteria for the nine selected items [2,11]. The four key items are indicated with two asterisks (**). All items were scored on a scale of one to seven, with one being “strongly disagree” and seven being “strongly agree.”

**Item 4: The guideline group includes individuals from all relevant professional groups**

Description

This item refers to the professionals who were involved at some stage of the development process. This may include members of the steering group, the research team involved in selecting and reviewing/rating the evidence and individuals involved in formulating the final recommendations. This item excludes individuals who have externally reviewed the guideline. This item excludes target population representation. Information about the composition, discipline, and relevant expertise of the guideline development group should be provided.

How to Rate

- For each member of the guideline development group, the following information is included:
  - Name
  - Discipline/content expertise
  - Institution
  - Geographical location
  - Description of the member’s role in the guideline development group
- Additional considerations:
  - Is the item well written? Are the descriptions clear and concise?
  - Is the item content easy to find in the guideline?
  - Are the members an appropriate match for the topic and scope? Potential candidates include relevant clinicians, content experts, researchers, policy makers, clinical administrators, and funders.
  - Is there at least one methodology expert included in the development group (e.g., systematic review expert, epidemiologist, statistician, library scientist, etc.)?

**Item 7: Systematic methods were used to search for evidence.**

Description

Details of the strategy used to search for evidence should be provided including search terms used, sources consulted, and dates of the literature covered. Sources may include electronic databases (e.g. MEDLINE, EMBASE, CINAHL), databases of systematic reviews (e.g. the Cochrane Library, DARE), handsearching journals, reviewing conference proceedings, and other guidelines (e.g. the US National Guideline Clearinghouse, the German Guidelines Clearinghouse). The search strategy should be as comprehensive as possible and executed in a manner free from potential biases and sufficiently detailed to be replicated.

How to Rate

- Item content includes the following criteria:
  - Named electronic database(s) or evidence source(s) where the search was performed
  - Time periods searched
  - Search terms used
  - Full search strategy included
- Additional considerations:
  - Is the item well written? Are the descriptions clear and concise?
  - Is the item content easy to find in the guideline?
  - Is the search relevant and appropriate to answer the health question?
  - Is there enough information provided for anyone to replicate the search?

**Item 8: The criteria for selecting the evidence are clearly described.**

Description

Criteria for including/excluding evidence identified by the search should be provided. These criteria should be explicitly described and reasons for including and excluding evidence should be clearly stated. For example, guideline authors may decide to only include evidence from randomized clinical trials and to exclude articles not written in English.

How to Rate

- Description of the inclusion criteria, including:
  - Target population characteristics
  - Study design
  - Comparisons (if relevant)
  - Outcomes
  - Language (if relevant)
  - Context (if relevant)
- Description of the exclusion criteria (if relevant)
- Additional considerations:
  - Is the item well written? Are the descriptions clear and concise?
  - Is the item content easy to find in the guideline?
  - Is there a rationale given for the chosen inclusion/exclusion criteria?
  - Do inclusion/exclusion criteria align with the health question(s)?
  - Are there reasons to believe that relevant literature may not have been considered?

**Item 10:** **The methods for formulating the recommendations are clearly described.**

Description

A description of the methods used to formulate the recommendations and how final decisions were arrived at should be provided. For example, methods may include a voting system, informal consensus, and formal consensus techniques (e.g., Delphi, Glaser techniques). Areas of disagreement and methods of resolving them should be specified.

How to Rate

- Item content includes the following criteria:
  - Description of the recommendation development process
  - Outcomes of the recommendation development process
  - Description of how the process influenced the recommendations
- Additional considerations:
  - Is the item well written? Are the descriptions clear and concise?
  - Is the item content easy to find in the guideline?
  - Was a formal process used to arrive at the recommendations?
  - Were the methods appropriate?

**Item 12: There is an explicit link between the recommendations and the supporting evidence.**

Description

An explicit link between the recommendations and the evidence on which they are based should be included in the guideline. The guideline user should be able to identify the components of the body of evidence relevant to each recommendation.

How to Rate

- Item content includes the following criteria:
  - The guideline describes how the guideline development group linked and used the evidence to inform recommendations.
  - Each recommendation is linked to a key evidence description/paragraph and/or reference list.
  - Recommendations linked to evidence summaries, evidence tables in the results section of the guideline.
- Additional considerations:
  - Is there congruency between the evidence and recommendations?
  - Is the link between the recommendations and supporting evidence easy to find in the guideline?
  - When evidence is lacking or a recommendation is informed primarily by consensus of opinion by the guideline group, rather than the evidence, is this clearly stated and described?

**Item 13: The guideline has been externally reviewed by experts prior to its publication.**

Description

A guideline should be reviewed externally before it is published. Reviewers should not have been involved in the guideline development group. Reviewers should include experts in the clinical area as well as some methodological experts. Target population (patients, public) representatives may also be included. A description of the methodology used to conduct the external review should be presented, which may include a list of the reviewers and their affiliation.

How to Rate

- Item content includes the following criteria:
  - Purpose and intent of the external review
  - Methods taken to undertake the external review
  - Description of the external reviewers
  - Outcomes/information gathered from the external review
  - Description of how the information gathered was used to inform the guideline development process and/or formation of the recommendations
- Additional considerations:
  - Is the item well written? Are the descriptions clear and concise?
  - Is the item content easy to find in the guideline?
  - Are the external reviewers relevant and appropriate to the scope of the guideline? Was there a rationale given for choosing the included reviewers?
  - How was information from the external review used by the guideline development group?

**Item 15: The recommendations are specific and unambiguous.**

Description

A recommendation should provide a concrete and precise description of which option is appropriate in which situation and in what population group, as informed by the body of evidence.

- An example of a specific recommendation is: Antibiotics should be prescribed in children two years or older with a diagnosis of acute otitis media if the pain lasts longer than three days or if the pain increases after the consultation despite adequate treatment with painkillers; in these cases, amoxicillin should be given for 7 days (supplied with a dosage scheme).
- An example of a vague recommendation is: Antibiotics are indicated for cases with an abnormal or complicated course.

It is important to note that in some instances, evidence is not always clear cut and there may be uncertainty about the best care option(s). In this case, the uncertainty should be stated in the guideline.

How to Rate

- Item content includes the following criteria:
  - Statement of the recommended action
  - Identification of the intent or purpose of the recommended action
  - Identification of the relevant population
  - Caveats or qualifying statements (if relevant)
- Additional considerations:
  - In the event of multiple recommendations (e.g., management guidelines), is there clarity regarding to whom each recommendation applies?
  - If there is uncertainty in the interpretation and discussion of the evidence, is the uncertainty reflected in the recommendations and explicitly stated?

**Item 22: The views of the funding body have not influenced the content of the guideline.**

Description

Many guidelines are developed with external funding (e.g., government, professional associations, charity organizations, pharmaceutical companies). Support may be in the form of financial contribution for the complete development, or for parts of it (e.g., printing of the guidelines). There should be an explicit statement that the views or interests of the funding body have not influenced the final recommendations.

How to Rate

- Item content includes the following criteria:
  - The name of the funding body or source of funding (or explicit statement of no funding)
  - A statement that the funding body did not influence the content of the guideline
- Additional considerations:
  - Is the item well written? Are the descriptions clear and concise?
  - Is the item content easy to find in the guideline?
  - How did the guideline development group address potential influence from the funding body?

**Item 23: Competing interests of guideline development group members have been recorded and addressed.**

Description

There are circumstances when members of the development group may have competing interests. For example, this would apply to a member of the development group whose research on the topic covered by the guideline is also funded by a pharmaceutical company. There should be an explicit statement that all group members have declared whether they have any competing interests.

How to Rate

- Item content includes the following criteria:
  - Description of the types of competing interests considered
  - Methods by which potential competing interests were sought
  - Description of the competing interests
  - Description of how the competing interests influenced the guideline process and development of recommendations
- Additional considerations:
  - Is the item well written? Are the descriptions clear and concise?
  - Is the item content easy to find in the guideline?
  - What measures were taken to minimize the influence of competing interests on guideline development or formulation of the recommendations?

**APPENDIX A4**

An adapted version of the spreadsheet utilized for data extraction can be found below.

**Guideline Information**

- Full reference
- Title of guideline
- Authors/professional organization
- Professional organizations’ website
- Year of publication
- Country and WHO region of publication
- Access: Where the guideline, its appendices, and other related documents can be accessed

**Scope and Purpose**

- Target population: The primary population(s) addressed by the recommendation(s) in the guideline
- Diseases/conditions covered: Ear and hearing conditions covered by the guideline
- Key objectives: The objective(s) of the guideline, such as improvements in health indicators (e.g., disease prevalence, quality of life, or cost savings)
- Target audience or end users
- Clinical conditions/questions addressed
- Setting: Setting(s) of the first contact at the clinical level (primary, secondary, tertiary) for which the guideline is intended.

**Methodology**

- Guideline development groups: How all contributors to the guideline development were selected and their roles/ responsibilities (e.g., steering group, guideline panel, external reviewer, systematic review team, methodologists)
- Process of guideline development
- Strength of recommendations (validation process)
- Year of original guideline publication
- Year(s) of subsequent revised guideline(s)

**Content of Guideline**

- Treatment protocols
- Diagnostic procedures
- Implementation tools

**Review and Quality Assurance**

- External review: Whether the draft guideline underwent independent review, and if so, how this was executed and the comments considered and addressed
- Quality assurance processes
- Limitations of guideline

**REFERENCES**:

1. Dillard LK, Mishra P, Der C, Chadha S. Protocol for the development of the WHO package of ear and hearing care interventions. BMJ Public Health 2025;3(1): e002852.

2. Brouwers MC, Kerkvliet K, Spithoff K, Consortium ANS. The AGREE Reporting Checklist: a tool to improve reporting of clinical practice guidelines. BMJ 2016;354: i4852.

3. Keel S, Evans JR, Block S, Bourne R, Calonge M, Cheng C et al. Strengthening the integration of eye care into the health system: methodology for the development of the WHO package of eye care interventions. BMJ Open Ophthalmol 2020;5(1): e000533.

4. Rauch A, Negrini S, Cieza A. Toward Strengthening Rehabilitation in Health Systems: Methods Used to Develop a WHO Package of Rehabilitation Interventions. Arch Phys Med Rehabil 2019;100(11): 2205–11.

5. National Institute for Health and Care Excellence. Hearing loss in adults: assessment and management. <https://www.nice.org.uk/guidance/ng98> (2018). Accessed 5 Apr 2025.

6. Schwartz SR, Magit AE, Rosenfeld RM, Ballachanda BB, Hackell JM, Krouse HJ et al. Clinical Practice Guideline (Update): Earwax (Cerumen Impaction). Otolaryngol Neck Surg 2017;156(S1): S1–29.

7. Horton GA, Simpson MTW, Beyea MM, Beyea JA. Cerumen Management: An Updated Clinical Review and Evidence-Based Approach for Primary Care Physicians. J Prim Care Community Health 2020;11: 2150132720904181.

8. British Society of Audiology. Aural Care (Ear Wax Removal). <https://www.thebsa.org.uk/resources/> (2019). Accessed 8 Jun 2025.

9. Oron Y, Shushan S, Ben-David N, Flaksman H, Korenbrot F, Merrik J et al. Guidelines for Ear, Nose, and Throat Examination of Adults With Intellectual Disabilities: Report of a Clinical Practice Application. J Policy Pract Intellect Disabil 2015;12(4): 272–8.

10. Hauk L. Cerumen Impaction: An Updated Guideline from the AAO–HNSF. Am Fam Physician 2017;96(4): 263–4.

11. AGREE II Next Steps Consortium. The AGREE II Instrument. <http://www.agreetrust.org> (2017). Accessed 8 Jun 2025.
